# Assessment of perinatal outcome after sustained tocolysis in early labour (APOSTEL-II trial)

**DOI:** 10.1186/1471-2393-9-42

**Published:** 2009-09-09

**Authors:** Carolien Roos, Liesbeth HCJ Scheepers, Kitty WM Bloemenkamp, Annemiek Bolte, Jerome Cornette, Jan B Derks, Hans JJ Duvekot, Jim van Eyck, Joke H Kok, Anneke Kwee, Ashley Merién, Brent C Opmeer, Mariëlle G van Pampus, Dimitri NM Papatsonis, Martina M Porath, Joris AM van der Post, Sicco A Scherjon, Krystyne Sollie, Marc EA Spaanderman, Sylvia MC Vijgen, Christine Willekes, Ben Willem J Mol, Fred K Lotgering

**Affiliations:** 1 Department of Obstetrics and Gynaecology, Radboud University Nijmegen Medical Centre, Nijmegen, The Netherlands; 2 Department of Obstetrics and Gynaecology, Maastricht University Medical Centre, Maastricht, The Netherlands; 3 Department of Obstetrics and Gynaecology, Leiden University Medical Centre, Leiden, The Netherlands; 4 Department of Obstetrics and Gynaecology, VU Medical Centre, Amsterdam, The Netherlands; 5 Department of Obstetrics and Gynaecology, Erasmus Medical Centre, Rotterdam, The Netherlands; 6 Department of Obstetrics and Gynaecology, University Medical Centre, Utrecht, The Netherlands; 7 Department of Obstetrics and Gynaecology, Isala Clinics, Zwolle, The Netherlands; 8 Department of Neonatology, Academic Medical Centre, Amsterdam, The Netherlands; 9 Department of Obstetrics and Gynaecology, Máxima Medical Centre, Veldhoven, The Netherlands; 10 Department of Clinical Epidemiology, Biostatistics and Bioinformatics, Academic Medical Centre, Amsterdam, The Netherlands; 11 Department of Obstetrics and Gynaecology, University Medical Centre, Groningen, The Netherlands; 12 Department of Obstetrics and Gynaecology, Amphia Hospital, Breda, The Netherlands; 13 Department of Obstetrics and Gynaecology, Academic Medical Centre, Amsterdam, The Netherlands

## Abstract

**Background:**

Preterm labour is the main cause of perinatal morbidity and mortality in the Western world. At present, there is evidence that tocolysis for 48 hours is useful in women with threatened preterm labour at least before 32 weeks. This allows transfer of the patient to a perinatal centre, and maximizes the effect of corticosteroids for improved neonatal survival. It is questionable whether treatment with tocolytics should be maintained after 48 hours.

**Methods/Design:**

The APOSTEL II trial is a multicentre placebo-controlled study. Pregnant women admitted for threatened preterm labour who have been treated with 48 hours corticosteroids and tocolysis will be eligible to participate in the trial between 26^+0 ^and 32^+2 ^weeks gestational age. They will be randomly allocated to nifedipine (intervention) or placebo (control) for twelve days or until delivery, whatever comes first.

Primary outcome is a composite of perinatal death, and severe neonatal morbidity up to evaluation at 6 months after birth. Secondary outcomes are gestational age at delivery, number of days in neonatal intensive care and total days of the first 6 months out of hospital. In addition a cost-effectiveness analysis will be performed. Analysis will be by intention to treat. The power calculation is based on an expected 11% difference in adverse neonatal outcome. This implies that 406 women have to be randomised (two sided test, β 0.2 at alpha 0.05).

**Discussion:**

This trial will provide evidence as to whether maintenance tocolysis reduces severe perinatal morbidity and mortality in women with threatened preterm labour before 32 weeks.

**Trial Registration:**

Clinical trial registration: , NTR 1336, date of registration: June 3^rd ^2008.

## Background

Preterm birth is the most common cause of neonatal morbidity and death worldwide [1]. Two thirds of the preterm births occur as a result of spontaneous labour beginning with spontaneous contractions or with preterm rupture of membranes. Preterm birth accounts for approximately 75% of all neonatal deaths and 50% of childhood neurological morbidities [2]. Moreover, it is associated with high immediate and long-term costs after discharge from the hospital [3]. These include costs for special education services and institutionalised care for physically and mentally disabled infants [4]. The prevalence of adverse neonatal outcome is strongly related to gestational age at delivery and declines from 77% at 24-27 weeks to less then 2% at 34 weeks and beyond [5]. Perinatal death and morbidity are not only strongly related to early gestational age but also to whether or not antenatal corticosteroids are administered [6] and whether a preterm infant is transferred to a tertiary care centre before or after birth. Postponing delivery for 48 hours with tocolytics in order to allow maximal effect of maternal parenteral steroid administration and transfer of the mother to a centre with Neonatal Intensive Care Unit (NICU) facilities is therefore standard treatment in women with the diagnosis of threatened preterm labour before 32 weeks gestational age [7].

Approximately 75% of women with a diagnosis of threatened preterm labour have not delivered after the first 48 hours of tocolytic therapy with nifedipine. After this 48 hour period the risk of preterm delivery persists. Two weeks after treatment for threatened preterm labour with nifedipine 65% of women are still pregnant [8].

Fetal fibronectin can be helpful in selecting women at risk for preterm delivery. Management based on knowledge of fetal fibronectin results significantly reduces preterm delivery less than 37 weeks compared to management without knowledge of these results (RR 0.54, 95% confidence interval 0.34 to 0.87). However the incidence of preterm delivery less than 34, 32 and 28 weeks were similar in both groups [9].

In national and international guidelines, a uniform treatment of threatened preterm delivery after 48 hours of tocolytic therapy has not yet been developed. Some obstetricians maintain tocolytic therapy until term, 37 weeks gestational age, whereas others stop tocolytics after 48 hours irrespective of gestational age or continue until for example 28 weeks gestational age. Maintenance treatment with several tocolytic agents has been being carried out in daily practice and include betamimetics [10,11], magnesium sulphate, indomethacin and calcium channel blockers [12] in order to achieve further prolongation of pregnancy and improvement of neonatal outcome. At present, no beneficial effect on perinatal outcome of such prolonged treatment has been established [13]. On one hand, tocolytic maintenance therapy with calcium channel blockers might be beneficial due to a positive effect on gestational age and possibly on neonatal outcome. On the other hand, use of tocolytics is associated with rare but severe side effects on mother and child [14,15], and may increase the risk of some perinatal complications - including intra-uterine infection.

Two systematic reviews included in DARE [16,17] and the Cochrane systematic review on maintenance tocolysis with nifedipine [12] reported only a few studies on the subject, and no reliable statement on the effectiveness of tocolytic maintenance therapy (TMT) on neonatal outcome. Although nifedipine maintenance therapy may increase gestational age at delivery, there has been no improvement in neonatal or maternal outcome [18-20].

In summary, preterm delivery is an important health care problem. Whereas it is evident that tocolysis with administration of corticosteroids for 48 hours is effective, there is insufficient evidence for a uniform policy after these 48 hours. In this randomised clinical trial we will investigate the effectiveness of maintenance therapy with nifedipine compared to placebo therapy in women with a gestational age below 32 weeks. Additional data will be collected for post-hoc evaluation of the clinical relevance of cervical length measurement and the presence of fibronectin in cervical mucus prior to maintenance tocolytic therapy.

This study is conducted within the Dutch Obstetric Consortium, a collaborative effort of obstetric clinics in The Netherlands to perform clinical trials. All ten Dutch perinatal centres with NICU facilities will participate in this trial.

## Methods/Design

### Aims

The aim of this study is to evaluate the effectiveness of tocolytic maintenance therapy on perinatal outcome after initial standard 48-hours' tocolytic therapy in women with threatened preterm birth between 26^+0^-32^+2 ^weeks gestational age. The outcome is measured in terms of neonatal mortality and composite neonatal morbidity (chronic lung disease, severe intraventricular haemorrhage, periventricular leucomalacia, proven sepsis and necrotising enterocolitis), gestational age at delivery and costs.

### Participants/eligibility criteria

Women with threatened preterm delivery with a gestational age between 26^+0 ^and 32^+2 ^weeks who have not delivered after 48 hours of tocolytics and corticosteroids are eligible for participation in the APOSTEL II-trial. Women with both singleton and multiple gestations are included. We chose not to include women with a gestational age between 24 and 26 weeks to prevent protocol violation (rescue tocolysis during study medication).

Maternal exclusion criteria are signs of intrauterine infection, placenta praevia, maternal disease requiring delivery (i.e. HELLP syndrome or preeclampsia), maternal hypertension and contraindications for the use of nifedipine. Fetal exclusion criteria are signs of fetal distress (abnormal cardiotocogram, abnormal biophysical profile), serious congenital defects and intrauterine death.

### Procedures, recruitment, randomisation and collection of data

The research nurse and/or the staff of participating hospitals will identify eligible women. After the patient has given informed consent for participation in the study, she is randomised using an internet-based procedure. Randomisation is 1:1 for nifedipine or placebo.

At study entry baseline demographics, obstetric and medical history are recorded. For post-hoc analysis on a subset of women, at study entry cervical length is measured by transvaginal ultrasound and a vaginal swab is taken for fibronectin testing. Fibronectin will not be tested in women with ruptured membranes, more than 3 cm dilatation or vaginal bleeding. Fibronectin swabs are stored and analyzed after the patient has delivered. All data are collected, coded and processed with adequate precautions to ensure patient confidentiality.

### Interventions

Patients are allocated to nifedipine or placebo for twelve days. Start of study medication is 48 hours after start of the initial tocolysis. Initial tocolysis is provided according to local protocol, usually this will be Nifedipine or Atosiban. Study medication consists of 20 milligrams nifedipine every six hours, administered orally, resulting in a total daily dose of 80 milligrams, or placebo. The medication is phased out from day 10 (total daily dose 60 milligrams nifedipine) till day 12 (total daily dose 20 milligrams nifedipine) and discontinued on day 13.

After randomisation the medication package is stored by the patient herself. The administration of the study medication is noted in a schedule that is kept both by the patient and in her medical record. Non-compliance is defined as a delay in administration of studymedication of more than 6 hours.

### Follow up of women and infants

All details of delivery, maternal assessments and admissions during pregnancy are recorded in an electronic case report form (CRF). Details of neonatal admissions are also recorded. Long-term follow up of children is depending on future funding.

### Outcome measures

The primary outcome measure is neonatal mortality and a composite of neonatal morbidity. The composite morbidity rate contains chronic lung disease, severe intraventricular haemorrhage more than grade 2, periventricular leucomalacia more than grade 1, proven sepsis and necrotising enterocolitis at six months after birth.

Secondary outcome measures are gestational age at delivery, birth weight, days on supported ventilation and additional oxygen, length of admission in neonatal intensive care, total days in hospital until three months corrected age and costs. Moreover, we will compare the number of days that each neonate surpasses outside the hospital within the first 6 months after the calculated term date.

### Statistical issues

#### Sample size

The sample size is calculated based on an 11% reduction in the primary outcome 'composite neonatal morbidity', from 25% to 14%. Using a two-sided test with an alpha of 0.05 and a power of 0.80 we have to randomise 406 patients (203 in each arm).

#### Data analysis

Data will initially be analysed according to the intention-to-treat method. First, the nifedipine and placebo groups will be compared. Relative risks and 95% confidence intervals will be calculated for the relevant outcome measures.

Planned subgroup analysis will be performed to assess the consistency of a treatment effect among various patient characteristics i.e. cervical fibronectin status, presence of ruptured membranes, multiple pregnancy as well as cervical length at study entry. We will test for interaction between these characteristics and treatment effect.

#### Interim analysis

An interim analysis will be performed after the follow up data of 100 and 200 women have been obtained. The analyses will be done by an independent Data and Safety Monitoring Committee (DSMC) that is not aware of treatment allocation when they judge the data on effectiveness. In case of patients reporting severe side-effects, the DSMC can order to disclose the label of these patients.

### Economic evaluation

#### General considerations

The aim of the economic evaluation is to compare the optimality, in terms of costs and health effects, of maintenance tocolysis with nifedipine versus placebo. As the clinical study is based on a superiority design (it is hypothesized that nifedipine decreases preterm birth), the proper economic evaluation design is a cost-effectiveness analysis (CEA): the optimal strategy will probably be dominant, i.e. better health outcomes and lower costs. The economic evaluation will be performed from a societal perspective.

#### Cost analysis

The process of care is divided into three cost stages (antenatal stage, delivery/childbirth, postnatal stage) and three cost categories (direct medical costs, direct non-medical costs and indirect costs). For each stage and each cost category, costs are measured as the volumes of resources used multiplied by appropriate valuations (cost-per-unit estimates, fees, national reference prices).

Volumes of health care resource use are measured prospectively alongside the clinical study in all participating centres as part of the CRF.

Valuations of direct medical resources (unit costs) are estimated comprising "true economic" costs, i.e. including shares of fixed costs and hospital overheads. An analysis based on reimbursement fees is added. Direct medical resources used outside the hospital and direct non-medical volumes are valued using national reference prices. Indirect costs are quantified but remain unvalued. Study-specific costs are excluded from analysis.

### Ethical consideration

This study has been approved by the ethics committee of the Academic Medical Centre Amsterdam (Ref. no. MEC 07/286) and by the boards of management of all participating hospitals. The trial is registered in the Dutch Trial Register, NTR 1336, , date of registration: June 3^rd ^2008.

## Discussion

Preterm birth is responsible for approximately 75% of all neonatal deaths and 50% of childhood neurological morbidities [2]. It is also associated with high immediate and long-term costs after discharge from the hospital [3].

To date, it is not clear whether prolonged treatment with nifedipine is effective in reducing adverse perinatal outcome [12,13]. On the one hand, tocolytic maintenance therapy with calcium channel blockers may be beneficial due to its positive effect on gestational age. On the other hand, use of tocolytics is associated with rare but severe side effects on mother and child [14,15]. Moreover, prolongation of pregnancy may also increase the chance for some perinatal complications such as infection.

As far as we know, there are no similar ongoing studies that will report on the subject. Neither the ISRTCN index of trials (UK), nor IMPACT/PSANZ Perinatal Trials Registry (Australian) or NIH Clinical Trial database (USA) report any trials regarding maintenance tocolysis. There is one ongoing study (NIFTY study) registered that compares oral nifedipine with placebo, in women with singleton pregnancies between 24 and 34 weeks, with intact membranes and a positive fibronectin test, in whom a full course of corticosteroids has been completed. Primary outcome is prolongation of pregnancy for at least seven days. Secondary outcomes are duration and number of NICU admissions, and maternal and neonatal hospital costs. In this study, the sample size needed to detect a difference in neonatal morbidity or mortality between the groups was not calculated.

## Competing interests

The authors declare that they have no competing interests.

## Authors' contributions

JAP, FKL and BWJM were involved in conception and design of the study. CR, JAP, FKL, BWJM, MEAS and HCJS drafted the manuscript. All authors mentioned in the manuscript are members of the APOSTEL II study group. They participated in the design of the study during several meetings and are local investigators in the participating centres. All authors edited the manuscript and read and approved the final draft.

## Pre-publication history

The pre-publication history for this paper can be accessed here:


